# Technology-enhanced weight-loss program in multiple-cat households: a randomized controlled trial

**DOI:** 10.1177/1098612X211044412

**Published:** 2021-10-21

**Authors:** Barr N Hadar, Kenneth J Lambrecht, Zvonimir Poljak, Jason B Coe, Elizabeth A Stone, Adronie Verbrugghe, Theresa M Bernardo

**Affiliations:** 1 Department of Population Medicine, Ontario Veterinary College, University of Guelph, ON, Canada; 2 West Towne Veterinary Center, Fit Pets for Rescues, Madison, WI, USA; 3 Department of Clinical Studies, Ontario Veterinary College, University of Guelph, Guelph, ON, Canada

**Keywords:** Obesity management systems, ecosystem health monitoring, technology assisted weight loss, technology-enhanced weight loss, veterinary telemonitoring, veterinary medical technology, remote monitoring devices, weight loss programs, home pet health technology ecosystem, weight management technology

## Abstract

**Objectives:**

The objectives of this study were to determine whether a technology-enhanced weight-loss program, using a home pet health technology ecosystem, is an effective tool in feline weight-loss management in multiple-cat households and to evaluate its impact on cat behavior.

**Methods:**

The study was a prospective parallel unmasked block-randomized controlled trial comparing two weight loss intervention groups: (1) traditional group with dietary restriction alone (n = 9); (2) technology group that used dietary restriction, digital scales, smart feeders, activity monitors and pet treat cameras (n = 6). A 12-week weight-loss program of client-owned indoor-only two- or three-cat households with at least one overweight cat was conducted in Canada and the USA. Owner impressions of the technology, weight loss rates, smart feeder data, activity monitor data and health-related quality of life (HRQoL) were assessed.

**Results:**

The study was completed by 9/15 traditional group and 6/10 technology group cats. Dropouts were mainly due to owner issues unrelated to the study. The pet health technology ecosystem received favorable reviews (six responders). Smart feeders and home scales were perceived as valuable additions, while activity monitors and pet treat cameras were valued lower. The average weekly weight-loss rate (percent loss of initial body weight) was higher (*P* = 0.036) in the technology group (0.694%) than in the traditional group (0.175%). Although not associated with weight-loss rates, technology group cats trended toward grazing feeding patterns and decreased daily activity counts, while HRQoL increased, on average, for all cats.

**Conclusions and relevance:**

This introductory investigation suggests that a technology-enhanced weight-loss program would be accepted by cat owners and may deliver advantageous outcomes in multiple-cat households, providing an effective and practical tool in feline weight-loss strategies that will continue to evolve as new technologies become available. It also illustrates the potential value of data gathered from home monitoring devices and digital diaries, providing deeper insights into pet behavior.

## Introduction

Cat obesity is a significant problem, with an estimated prevalence of between 22% and 60% in different populations.^1–10^ Even after maturity, many cats continue to gain weight until 8–10 years of age.^11^ Obesity has detrimental effects on the health and longevity of pets, and is now classified as a disease by the Global Pet Obesity Initiative Position Statement.^12^ Excess body fat in cats predisposes to or is associated with many health-related conditions, including diabetes mellitus, orthopedic disease, heart disease, feline lower urinary tract disease, skin conditions and neoplasia.^1,2,13–19^

Feline weight-loss strategies usually involve strict dietary management, including a veterinary weight-loss diet and energy restriction, sometimes in conjunction with increased activity. Major barriers to effective pet weight management often include lack of owner recognition of their pet’s weight problem, insufficient client education on pet nutrition and weight management, inaccurate food measuring, and lack of follow-up visits for reassessment, feedback and coaching. Owner engagement and consistent veterinary supervision are vital to successful weight management, particularly in multiple-cat households with food stealing, a need for different foods and variable feeding styles. Recent reviews of human clinical trials have found that technology-assisted weight-loss interventions were advantageous compared with traditional methods.^20,21^

The primary objective of this study was to determine whether a home pet health technology ecosystem (PHTE), including a digital scale, smart feeders, activity monitors and a pet treat camera, was an effective tool in a feline weight-loss program (WLP) in multiple-cat households by comparing a traditional weight loss intervention, including a veterinary weight-loss diet and energy restriction, and a technology-enhanced PHTE weight-loss intervention. This study also aimed to evaluate health-related quality of life (HRQoL) and the impact of a smart feeder and activity monitor on feline behavior during a WLP.

## Materials and method

### Eligibility criteria

Inclusion criteria were as follows: body condition score (BCS) 7–8/9,^22,23^ 1–12 years of age, indoor-only, spayed/castrated, 2–3-cat household, acclimated to eating a dry diet, and no concurrent disease, condition, therapy or planned procedure that might influence the weight-loss process (based on the managing veterinarian’s discretion).

### Study protocol

The study was designed as a prospective, parallel, unmasked, randomized controlled trial comparing two weight-loss intervention groups (1:1 allocation ratio): (1) dietary restriction weight loss (traditional group) and (2) technology-enhanced dietary restriction weight loss (technology group). This study is reported according to the Consolidated Standards of Reporting Trials (CONSORT) statement guidelines with appropriate consent and approvals. Cat owners submitted their written consent to participate in the study and agreed to the use of their data for presentation and publication purposes. Approval was obtained from the University of Guelph’s Animal Care Committee (AUP #4101) and Research Ethics Board (REB# 19-01-038) for use of animal and human participants, respectively.

Convenience sampling of 4–6 veterinarians in Canada (private practices and the Ontario Veterinary College’s Smith Lane Animal Hospital at the Hill’s Pet Nutrition Primary Healthcare Centre, Guelph ON, Canada) and 4–6 veterinarians in the USA (private practices) was carried out based on relationships with the authors, known interest in feline nutrition and openness to technology. Each veterinarian was to recruit 2–4 households, whom the authors would enroll and randomize into intervention groups. A two-sample *t*-test power calculation was used to determine sample size. The minimum difference in weekly weight loss rate (WWLR) between groups (0.356%) and SD for both groups (0.5) were estimated based on results from recent cat weight-loss studies.^24–27^ Assuming a power of 80% and an alpha of 0.05, a target sample size of 64 cats (32 per group) was established. Owing to expected recruitment limitations, allocation of households to intervention groups was carried out by block randomization per veterinarian (block size of two) using a virtual coin flip (random.org). This would allow for near equal-sized intervention groups and intervention matching by veterinarian. The allocation process was determined by the authors and was unknown to the veterinarians and owners.

Veterinarians were instructed to perform an initial assessment of cats consisting of a physical examination and nutritional screening evaluation,^28^ with veterinary determination of body weight (BW), BCS, muscle condition score (MCS; World Small Animal Veterinary Association MCS Scale) and general health condition. Veterinarians were also asked to have a complete blood count and biochemistry panel on cat participants within the past 12 months (not collected by the research team), but ultimately this decision was left to the managing veterinarian’s discretion.

Once cat owners provided consent to participate in the study, the technology group households received a PHTE consisting of a pediatric scale (Comfort Baby Scale; Smart Weigh), one smart feeder per cat in the household (SureFeed Microchip Pet Feeder Connect; Sure Petcare), one activity monitor per cat in the household (FitBark 2 Activity Monitor; FitBark) and a pet treat camera (Petcube Bites; Petcube [Table 1; see also Table S1 in the supplementary material]). After PHTE set-up instructions were completed, cats and owners were given 2 weeks (weeks 1 and 2; days 0–14) of PHTE adjustment prior to the caloric restriction period. Traditional group households did not receive a PHTE but also waited 2 weeks (as a control) after initial examination to start the caloric restriction period.

**Table 1:** Pet health technology ecosystem: components and specifications

Device*	Specifications
Smart Weigh pediatric scale	Accurate to 0.01 kg/0.022 lb and validated by an electrical engineer Owners were asked to record body weights in a weekly diary by using the scale on a hard surface at a consistent daily time One per household
SureFeed Microchip Pet Feeder Connect	Patient controlled (microchip/ID tag) and portion controlled (built in gram scale with light visual to indicated correct amount of food) with associated smartphone application and ability to share cloud-based real-time data One per cat
Fitbark Activity monitor	Three-axis accelerometers with associated smartphone application and ability to share cloud-based real-time data. Readings taken multiple times per second and integrated over a 1 min epoch. Research-grade and comparable specifications to the validated Actical monitors but more widely available and affordable to pet owners Owners asked to place on collar of each cat One per cat
Petcube Bites pet treat camera	Motion/sound activated, night-vision enabled, web cameras with the ability to toss dry treats up to 6 feet through associated smartphone application Owners asked to point toward feeding stations One per household

* Devices were chosen based on proposed technological solutions that would facilitate delivery and implementation of weight loss strategies (Table S1 in the supplementary material). Specific devices in each category were chosen based on user and author preference established during a pilot study

Both groups followed a standard WLP that included a 1-week food adaptation period (week 2; days 8–14) followed by a 12-week caloric restriction period (weeks 3–14; days 15–98). Study protocol directed the use of the Pet Nutrition Alliance calculator (Adult Cat Calorie Calculator; Pet Nutrition Alliance) to estimate ideal BW (5% weight reduction for every half number above 5 on a 1–9 BCS scale) and caloric intake (0.8 * resting energy requirement, with resting energy requirement = 70 * [ideal BW]^0.75^), which is consistent with the 2014 American Animal Hospital Association weight management guidelines.^29^ The preferred method of calculating restriction is based on a reduction of pre-WLP caloric intake. However, this was not the method used for the study due to likely inaccuracies in owner-reported caloric intake from measuring inaccuracies, leftover food in the bowl and food sharing/stealing issues in multiple-cat households. Veterinarians used gram scales (Stainless Steel Digital Kitchen Scale; Amazon Basics) to determine food volume and then provided marked food measuring cups to traditional group households. Food weight was controlled by use of a smart feeder in the technology group households.

**Figure 1:**
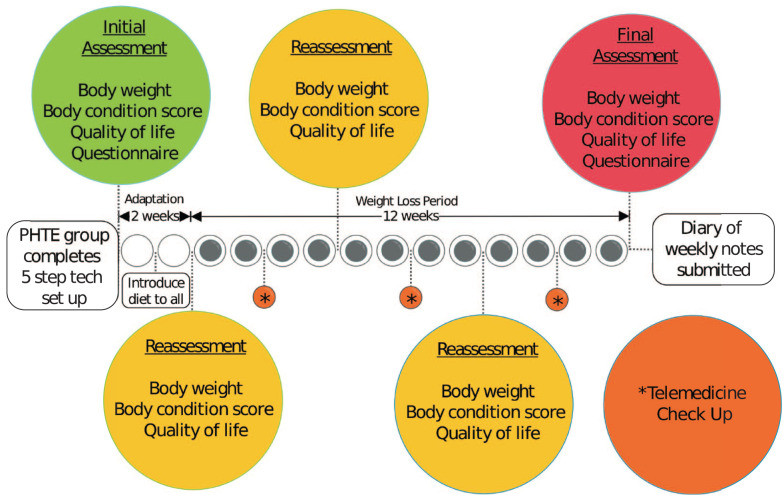
Study timeline. PHTE = pet health technology ecosystem

All households received a prescription weight-loss diet (Metabolic Feline Dry; Hill’s Pet Nutrition) to be fed three times daily, allocating at least 90% of caloric intake to the prescribed food and at most 10% of caloric intake for other food, including wet food and treats. Recheck appointments were recommended at weeks 2, 6, 10 and 14 and telemedicine check-ups (email, text, phone, video, etc) at weeks 4, 8 and 12 to ensure adequate and safe weight loss (Figure 1). Telemedicine check-ups for traditional group households served as a time to discuss issues or concerns, as BWs were not collected at home. The target WWLR was 1% with a recommendation to decrease caloric intake by 10% if WWLR <0.5% and increase caloric intake by 10% if WWLR >1.5%.

All owners were asked to complete pre-/post-WLP questionnaires online (Qualtrics CoreXM; Qualtrics) for their impressions (see the supplementary material), a weekly diary for WLP notes, cat HRQoL assessments (VetMetrica; NewMetrica)^30–32^ throughout the study period (initial, weeks 2, 6, 10 and 14), and a human–animal bond (HAB) assessment (Lexington Attachment to Pets Scale).^33,34^ Table 2 shows how the results were calculated.

**Table 2:** Outcome and description of calculation

Outcome	How it was calculated
Owner impressions	Online pre-/post-WLP questionnaires consisting of a combination of open-ended, closed, multiple choice and Likert-scale questions regarding cat weight loss history, WLP and PHTE experience, and cat behavior observations
Owner-perceived value of PHTE/devices	Net promoter scores were calculated for each device and the whole PHTE. Net promoter score is a widely used customer loyalty and satisfaction metric, based on how likely customers are to recommend the product to others (1–10 scale). Positive scores reflect more ‘promoters’ and negative scores reflect more ‘detractors’
WLP notes	A weekly diary consisting of notes by owners on cat body weight, cat diet (food and treats), cat behaviors, PHTE comments (technology group) and general comments
Amount eaten per meal, meal duration, daily number of meals	Smart feeder data: cat identification (microchip or ID tag), date/time, change in weight (g), ‘pet feed’ vs ‘owner top off’, and duration (s)
Activity	Activity monitor data: activity counts, calculated from three-dimensional accelerometer readings taken multiple times per second and integrated over a 1 min epoch
HRQoL	VetMetrica; a validated standardized questionnaire, answered by owners and used to calculate three domains scores (0–70 scale): vitality, comfort and emotional wellbeing. The average healthy cat has domain scores of 50, and 70% of healthy cats will score >44.8. Designed to minimize respondent bias, and assessments were spaced out to decrease memorization of answers by owners
Human–animal bond	Lexington Attachment to Pets Scale; a widely used instrument to assess human emotional attachments to pets and is composed of 23 Likert-scale questions regarding one owner and their favorite pet. This was then applied to all pets in household. Scores totaled for a maximum of 69 (strongest human–animal bond)
Percent weight loss	([Initial body weight – final body weight]/initial body weight) × 100
Average WWLR	(Percent weight loss/days of caloric restriction) × 7
Weight loss period	From the start of caloric intake restriction to their last clinic weigh-in

WLP = weight loss program; PHTE = pet health technology ecosystem; HRQoL = health-related quality of life; WWLR = weekly weight loss rate

### Statistical analyses

#### Devices and tools: feeding, activity, HRQoL and HAB

Amount eaten per meal, meal duration, daily number of meals, activity, HAB and HRQoL were analyzed using mixed-effects linear regression models to assess the following predictor variables: days of caloric restriction, age, sex, initial BCS, initial HRQoL scores, average WWLR, interaction of intervention group with the other variables and HAB (HRQoL only). Feeding and activity data were only analyzed for cats in the technology group using the PHTE devices.

#### Weight loss

The Mann–Whitney U-test was used to assess the average WWLR between intervention groups. In addition, univariable linear regression models were used to assess the association of average WWLR with the following predictor variables: intervention group, HAB, age, sex, initial BW, initial BCS and initial HRQoL. Multivariable linear regression models were used to assess for confounding and interaction of HAB and initial BCS on intervention group. Two separate mixed-effects linear regression models were also used to assess weight loss (average WWLR and individual measurements over time), treating households and cats as random effects, to account for clustering within households and repeated measures.

All statistical analyses were performed in R 4.0.0 using packages ‘lm’ and ‘lme’ for regression models.

## Results

**Figure 2:**
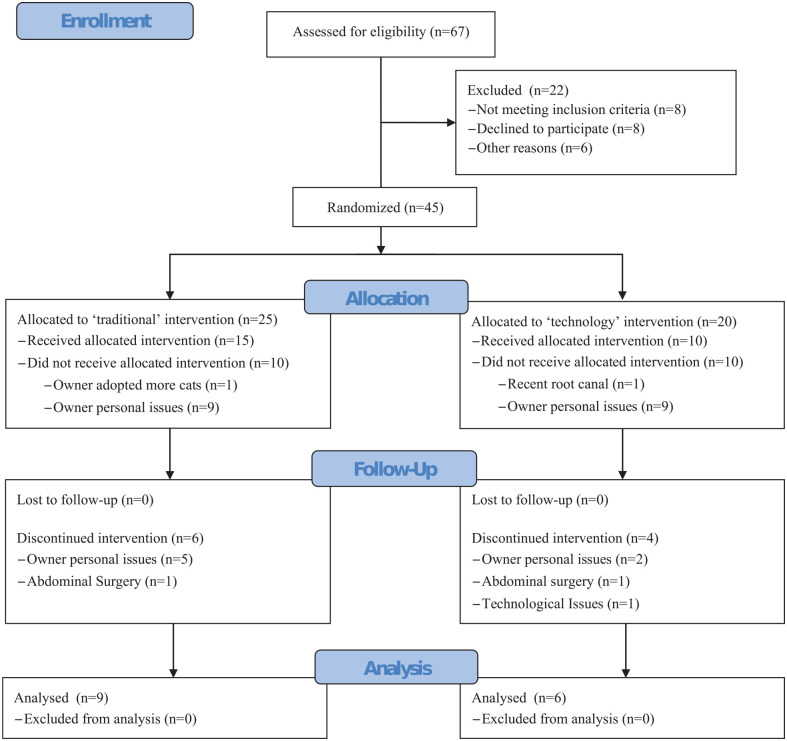
Consolidated Standards of Reporting Trials (CONSORT) flow diagram

Participants were recruited between February and August 2019, and data were collected through February 2020 (Table 3). Dropouts were mainly from owners deciding not to continue with the WLP for personal reasons, and none was lost to follow-up after study completion (Figure 2). The study was stopped before achieving the target sample size owing to logistical challenges (ie, difficult recruitment, time, resources). To incorporate more owner feedback, all submitted questionnaires were assessed, even if their cats were excluded from the rest of the analysis.

**Table 3:** Study population descriptive statistics

Item	Description
Total dropouts	30
Analyzed: weight loss, feeding, activity, HRQoL and HAB	Nine traditional group cats and six technology group cats completed the WLP
Final traditional group	Five traditional group households (nine cats; age 7.2 ± 3.2 years; initial weight 6.49 ± 1.34 kg; BCS 7.4 ± 0.5/9; breed DSH/DLH, all two-cat households)
Final technology group	Four technology group households (six cats; age 8.3 ± 0.8 years; initial weight 6.17 ± 1.26 kg; BCS 7.3 ± 0.5/9; breed DSH, one three-cat household)
Analyzed: owner impression	Thirteen pre-WLP (seven traditional/six technology) and 10 post-WLP (four traditional/six technology) questionnaires completed
Owner demographics	Age (range 20–60 years [mean 34]), sex (three males, 10 females) and country (seven from Canada, six from the USA).

HRQoL = health-related quality of life; HAB = human–animal bond; WLP = weight-loss program; BCS = body condition score; DSH = domestic shorthair; DLH = domestic longhair

### Owner impressions

All owners reported that they were proficient or better in technology use, perceived their cat(s) to be overweight, and many had previous experience with common weight-loss strategies, challenges and home pet devices (see Table S2 in the supplementary material). All owners strongly or somewhat agreed to the importance of ideal weight in their cats. There was high satisfaction (Likert-scale scores) with the WLP (see Table S3 in the supplementary material) and the PHTE (Table 4). Owners viewed the smart feeder as the most valuable, the home scale as valuable, and the activity monitor and pet treat camera with lower value as part of a WLP. Owners’ written comments highlighted the strengths and weaknesses of the PHTE used in the study (see Table S4 in the supplementary material). All owners would continue to use a PHTE in conjunction with a feline WLP but would not be willing to pay the approximate retail cost of $680 for the full set or $480 for the scale and two smart feeders only.

**Table 4:** Owner impressions of the pet health technology ecosystem (PHTE; n = 6)

Item	Owner response (number of owners)
Satisfaction with PHTE	Extremely satisfied (4), moderately satisfied (2)
PHTE effectiveness in a feline WLP	Extremely effective (3), very effective (3)
Would continue to use PHTE as part of WLP	Yes (6)
Willing to pay for PHTE	<$100 (3), $100–200 (2), $200–300 (1)
Net promoter score* of PHTE	80; excellent
Net promoter score of smart feeder	80; excellent
Net promoter score of home scale	0; decent
Net promoter score of activity monitor	–40; needs improvement
Net promoter score of pet treat camera	–60; needs improvement
Satisfaction with smart feeder and home scale	High Likert-scale satisfaction scores in all subcategories including device design, device set up, device use, accuracy and usefulness
Satisfaction with activity monitor	Lower satisfaction scores in usefulness and accuracy
Satisfaction with pet treat camera	Lower satisfaction scores in usefulness

* Net promoter score is a widely used customer loyalty and satisfaction metric. Scores range from −100 to 100 and are based on how likely customers are to recommend the brand or product to others (1–10 scale). Positive scores reflect more ‘promoters’ and negative scores reflect more ‘detractors’

WLP = weight-loss program

### Devices and tools: feeding, activity, HRQoL and HAB

**Figure 3:**
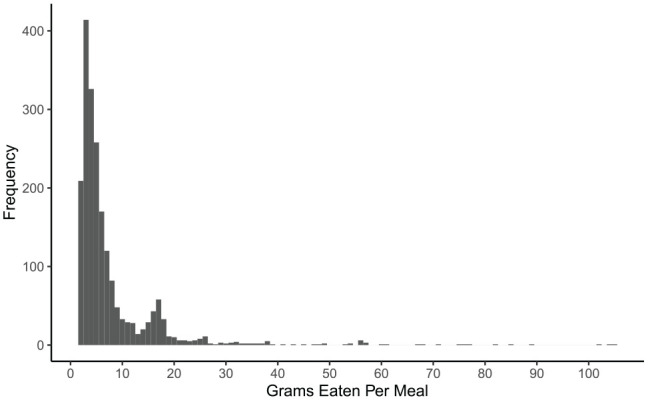
Histogram of meal amount (g) for cats using smart feeders

**Figure 4:**
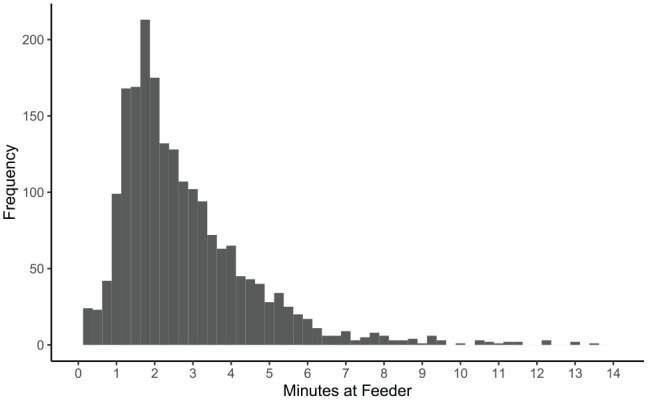
Histogram of meal duration (mins) for cats using smart feeders

**Figure 5:**
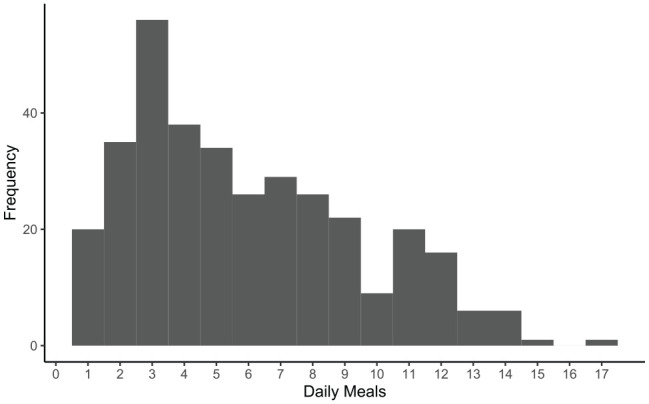
Histogram of the number of daily meals for cats using smart feeders

Average amount per meal (Figure 3) decreased over time (*P* <0.01), while average meal duration (Figure 4) increased slightly over time (*P* <0.01). Average number of daily meals (Figure 5) increased slightly over time but was not statistically significant (*P* = 0.245). Feeding behaviors were not significantly affected by age, sex, initial BCS, initial HRQoL or average WWLR. See Table 5 for specific smart feeder results.

**Table 5:** Feeder results

Result	Description
Meal amount	2–104 g and averaged 7.8 g (SD 9.56, median 4.75, IQR 2.42–7.08) Average amounts were 8.7 g, 7.9 g, 9.2 g and 5.9 g for quarter 1 (weeks 3–5), quarter 2 (weeks 6–8), quarter 3 (weeks 9–11) and quarter 4 (weeks 12–14), respectively
Meal duration	2 s to 13 mins and averaged 2.8 mins per meal Average times were 2.5 mins, 2.6 mins, 3.2 mins and 3.1 mins for quarters 1, 2, 3 and 4, respectively
Number of daily meals	1–17 and averaged 5.9 (SD 3.5, median 5, IQR 2.5–7.5) Average number of meals were 6.1, 6.2, 4.8 and 6.4 for quarters 1, 2, 3 and 4, respectively
Time of day of meals	Cats visited the feeder to eat at every hour of the day, with average peaks at 3 am, 10 am, 4 pm and 7 pm
Filling feeder frequency	Most owners filled feeders twice daily, followed by three times, one time and four times
Cat reaction to feeder	Some cats were initially hesitant when first introduced to the smart feeder, but all became comfortable with it within the 2-week adjustment period

Outliers and data obtained outside the weight-loss program period were excluded from analysis

Extreme/unreasonable values were assessed and appeared to be device artefacts, meal defined as feeder sessions designated as ‘pet feed’, with a negative change in weight between 2 and 105 g, and between 2 s and 1 h

IQR = interquartile range

**Figure 6:**
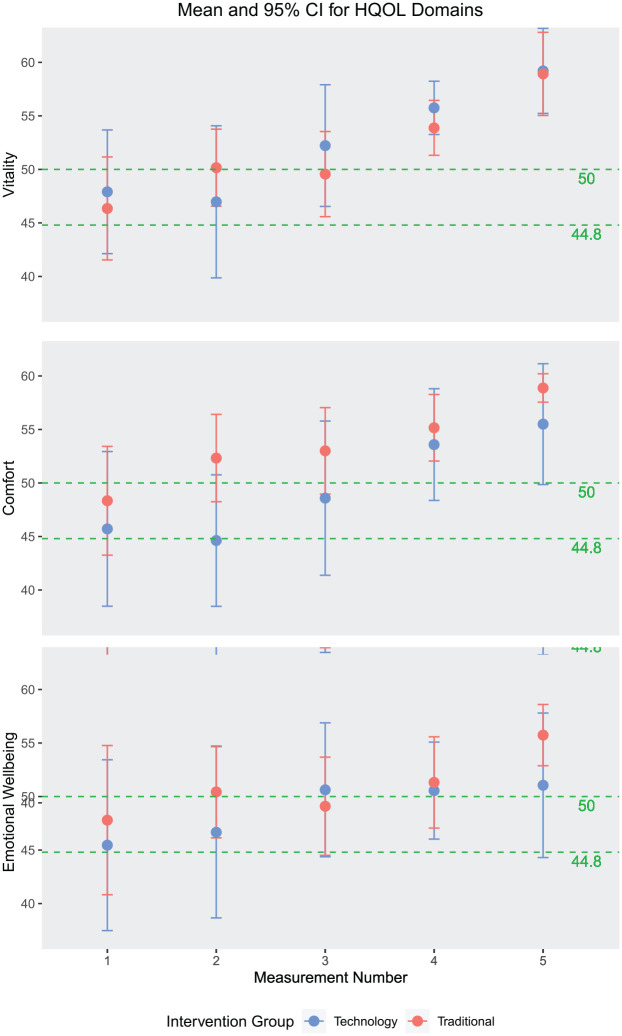
Mean and 95% confidence interval (CI) for health-related quality of life (HRQoL) domain scores. The average healthy cat had a score of 50 and 70% of healthy cats will score above the 44.8 threshold

Daily activity counts (mean 3809.1) decreased slightly over time (*P* <0.1) and with higher initial HRQoL scores: vitality (–55.1 ± 33.9; *P* = 0.049), comfort (–68.0 ± 15.8; *P* fn5-1098612<0.01) and emotional wellbeing (–47.5 ± 24; *P* = 0.031). Age, sex, initial BCS and average WWLR did not have a significant effect on daily activity counts. Some owners reported that the pet treat camera revealed unknown cat behaviors such as night playing, night eating and outsmarting the feeders (often due to improper set up). For all cats, HRQoL domain scores significantly (*P* <0.01) increased over time (Figure 6), increasing less with higher initial domain scores (*P* <0.01), eventually reaching similar values at the end of the study. Intervention group, HAB, age, sex, initial BCS and average WWLR did not have a significant effect on HRQoL domain scores. Lexington Attachment to Pets Scale (LAPS) scores were assessed for all but one cat (technology group), ranging from 31 to 65/69 and averaging 52.2 in both intervention groups.

### Weight loss

**Figure 7:**
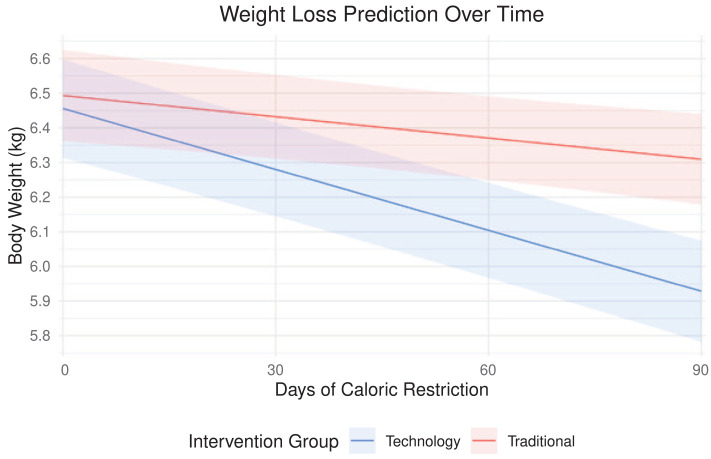
Expected weight change and 95% confidence band for a 90 day weight-loss program for an average cat in two intervention groups. This is based on the mixed-effects linear regression model from Table 6 with a starting body weight of 6.5 kg and all variables being equal

Weight loss was achieved in 5/9 cats in the traditional group that completed the study and all six cats in the technology group that completed the study. The statistical models used to assess weight loss rates by intervention group are presented in Table 6. Average WWLR was significantly higher in the technology group (0.694%) compared with the traditional group (0.175%) when assessed with the Mann–Whitney U-test (*P* = 0.036). Influence statistics did not indicate any major influence by household on the simple linear regression model. After accounting for HAB and initial BCS, a significant difference in average WWLR between intervention groups was still observed. A mixed-effects linear regression model, used to account for household clustering, did not reveal a significant difference in average WWLR between intervention groups (*P* = 0.089). However, another mixed-effects linear regression model did reveal a significant difference (*P* <0.01) in individual measurements over time (change in g/day) between intervention groups (Figure 7). Higher HAB led to slightly lower weight loss rates (–0.024 ± 0.021; *P* = 0.046). Age, sex, initial BW, initial BCS and initial HRQoL did not have a significant effect on average WWLR. MCS was not analyzed due to data limitations. Average initial BCS, LAPS scores and initial HRQoL were similar between intervention groups (Table 7).

**Table 6:** Weight loss rates by intervention group: statistical analysis

	Simple linear regression model		Multivariable linear regression model		Mixed-effects linear regression model		Mixed-effects linear regression model*	
	Coefficient (SE)	*P* value	Coefficient (SE)	*P* value	Coefficient (SE)	*P* value	Coefficient (SE)	*P* value
Intercept	0.694 (0.165)	<0.01	2.45 (0.81)	0.011	0.73 (0.19)	<0.01	348.75 (178.70)	0.055
Traditional	–0.519 (0.213)	0.030	–0.49 (0.20)	0.033	–0.51 (0.26)	0.089	37.60 (102.17)	0.724
LAPS	–	–	–0.02 (0.01)	0.046	–	–	–	–
Days of caloric restriction	–	–	–	–	–	–	–5.86 (0.58)	<0.01
Initial BW (kg)	–	–	–	–	–	–	939.54 (26.60)	<0.01
Days of caloric restriction: traditional (interaction)	–	–	–	–	–	–	3.82 (0.86)	<0.01
Household variance	–	–	–	–	0.33	–	13,417	–
Cat variance^†^	–	–	–	–	–	–	4650	–
Residual variance	–	–	–	–	–	–	17,261	–
Outcome	Average WWLR		Average WWLR		Average WWLR		g/day	

* Graphical representation in Figure 7

† Variance of random intercepts

LAPS = Lexington Attachment to Pets Scale (0–69; higher score = stronger owner attachment to pet); BW = body weight; WWLR = weekly weight-loss rate (%)

**Table 7:** Average caloric restriction duration, weight loss rates, initial body condition score (BCS), Lexington Attachment to Pets Scale (LAPS) and initial health-related quality of life (HRQoL) domain scores by intervention group

Treatment group	CR days	Average WWLR	Initial BCS	LAPS	Initial + Δ vitality *	Initial + Δ comfort *	Initial + Δ EWB *
Traditional	92.8	0.175	7.44	52.2	44.4 + **11.9**	47.6 + **9.7**	49.2 + 4.9
Technology	88	0.694	7.33	52.2	41.9 + **15.3**	42.4 + **12.6**	41.2 + **10.8**

* Health-related quality of life domain score by VetMetrica (0–70; 70% of healthy cats will score >44.8); Δ = change from initial score to final score; bold = improvement of 5 + for vitality or EWB and 7 + for comfort indicates a clinically meaningful change

CR days = caloric restriction days; WWLR = weekly weight-loss rate (target of 1%); BCS = body condition score (1–9); LAPS = Lexington Attachment to Pets Scale (0–69; higher score = stronger human–animal bond); EWB = emotional wellbeing

## Discussion

### Owner impressions

A major challenge faced by investigators studying technology is its rapid evolution. The optimal, and likely more affordable, PHTE will evolve as new technologies become available. The feeder appears to have addressed the top-rated challenge among study participants – food stealing between cats – while also providing consistent and accurate portioning. Participants also seem to have realized the value of continuously monitoring their cat’s weight with a home scale.

Now that affordable and accurate electronic scale technology is readily available, weight monitoring should be part of every cat owner’s routine, with a scale in every feline household to help with weight management and detect inadvertent weight loss. Ideally, this would be a passive ‘smart’ scale that could be placed in front of a feeder, under a bed or under a litter box. Recent litter box scales claim to activate automatically, recording not only body weight, but also – in some cases – urine/fecal output and cat elimination behaviors.^35,36^ However, these have yet to be validated for accuracy and cat-friendly design.

The potential advantage to owners of the activity monitor and pet treat camera may have been limited owing to the peripheral role they played in the WLP. Activity monitor value could have been increased by establishing a target change in activity counts, providing population benchmarks for comparison, and/or providing calorie expenditure calculations based on activity counts. There is currently very limited information regarding these in cats. The pet treat camera caught important feeding behaviors and allowed for remote engagement between owners and cats. While the ability of the treat-dispensing function to promote activity was limited and varied by cat, the device could be valuable for studying refined feeding behaviors.

### Devices and tools: feeding, activity and HRQoL

Cats in the technology group had smaller, longer meals throughout the WLP and did not have a significant change in meal frequency. However, changes in feeding patterns were not associated with weight loss rate. Perhaps this trend toward a grazing pattern is due to less inter-cat competition for food. This illustrates how data from smart feeders may improve the understanding of cat feeding behaviors. Our results are in contrast to a recent study, which found that cats had fewer, larger, quicker meals in response to caloric restriction.^37^ Unlike our study, these cats were not client owned, were housed in a research facility and had feeding patterns established through observation. Although owners were asked to feed three times daily, feeder data showed that owners fed 1–4 times a day, which may have influenced results and demonstrates the value of technology to help assess owner compliance. Results may have also been affected by accuracy of gram scales and data loss due to connectivity issues.

Daily activity counts in the technology group cats decreased slightly throughout the WLP, contrary to most owners’ perceptions (see Table S5 in the supplementary material). While accelerometers appear to be an effective tool for quantifying activity, studies have shown considerable inter-cat variability.^38,39^ This suggests that cats be used as their own controls when measuring activity counts. The ability of accelerometer data to distinguish locomotion vs other activity is another important consideration. Activity results may have been influenced by ‘inactive’ behaviors such as grooming and scratching, which are known to increase activity counts.^38,40^ Activity counts were not affected by weight loss rate or initial BCS. Weight gain in cats leads to a reduction in voluntary activity.^41,42^ Our results did not show an increase in voluntary activity with weight loss. This is consistent with other studies in cats and dogs. Average daily activity did not change significantly in another recent cat weight-loss study.^43^ Recent dog studies have shown that while obesity was associated with lower vigorous activity, weight loss was not associated with an increase in activity or reduction in sedentary behavior.^44,45^ Beyond weight loss, there are various researchers exploring the use of accelerometer data to gain insights into the behavior and health of pets. Recent investigations include the use of activity monitors to model jumping behavior and assess musculoskeletal impairment in cats.^46,47^ The ‘Pet Insight Project’ aims to create recognizable accelerometer profiles for common dog behaviors and medical conditions by combining information from accelerometers, observed behaviors and medical records.^48^

HRQoL increased throughout the WLP, but was not significantly affected by initial BCS or weight loss rate. No comparable studies were found in cats. However, this is consistent with a study that showed obese dogs had lower HRQoL scores that improved with successful weight loss, but was not associated with weight loss rate.^49,50^ Subjective scores for activity and quality of life also increased in another dog weight-loss study, without mention of weight loss rate association.^51^ While getting closer to ideal weight is thought to be associated with higher quality of life, hunger and other behaviors from rapid weight loss may be negatively perceived by owners.

### Weight loss

The fairly high dropout rate of this study confirms the difficulties of owner commitment to pet weight loss. This represents an opportunity to leverage technology to increase owner engagement and educate owners on the effects and costs associated with overweight pets. A technology-enhanced WLP with a PHTE delivered higher weight-loss rates than a standard WLP with traditional approaches alone. It should be noted that the average WWLRs were calculated using all cats, including four traditional group cats that gained weight. The typical target WWLR in overweight cats is 0.5–2%.^29^ Recent clinical studies in client-owned overweight cats had target WWLRs of 1–2% and showed average WWLRs of 0.38–0.8%.^24–27^ This is comparable to the technology group in this study, which had a lower target WWLR of 1% and involved multiple-cat households. Veterinary visits every 2–8 weeks were also performed in these studies to ensure adequate and safe weight loss, and to keeps owners engaged. A PHTE may allow for less frequent veterinary visits by empowering owners to take more control of their pets’ health and allowing veterinarians more frequent remote monitoring of their patients. Accurate information can be easily obtained in the home and frequent visits to the clinic can be avoided through the use of telemonitoring. This may improve the adherence to weight-management programs, given that most cats are averse to veterinary clinic visits. The value of a weekly digital diary should not be overlooked.

Weight loss may have been affected by differences in cats, households and veterinarians. However, there were no significant baseline differences apparent between intervention groups. Weight loss analysis included multiple statistical models that suggest household variability did not have a considerable impact on results. There was slight variation in veterinary management of cases, as this study was intended to reflect actual clinical practice. However, veterinarians were given instructions on caloric-intake calculations, appointment/communication timeline, PHTE set-up, and managed both a traditional and technology group for 6/9 households. This may have reduced variability between veterinarians, and ultimately households, but did not eliminate it. Caloric reduction amount compared with intake prior to the WLP was not calculated. Although unlikely, differences in this amount between intervention groups may have influenced weight loss rates.

### Limitations

Participation in this study was limited to convenience sampling of veterinary clinics in urban and suburban areas of Canada and the USA. Owing to the small sample size of this study, caution should be taken with the interpretation and generalizability of results. Significant differences in feeding behaviors, activity counts and HRQoL between the intervention groups may have been missed as this study contained a low number of observations and was likely underpowered for these outcomes. Two of the cats did not wear activity monitors for the entire study period and some feeder data were lost due to connectivity issues, further limiting the quantity and quality of device data. As ‘beta testers’ of these devices, we expected there to be some initial troubleshooting. However, this study illustrates the potential value of such devices. The PHTE was evaluated as a whole, and did not account for the effects of individual devices. Although our results suggest that the smart feeder and home scale had the biggest impact on weight loss, each device should be evaluated with separate intervention groups. Owing to time and resource limitations, this was not carried out in the current study. Further studies are also needed to explore longer term effects of a PHTE on weight management, including weight maintenance.

## Conclusions

This study serves as an introductory investigation, suggesting that a technology-enhanced WLP is accepted by owners and may deliver better outcomes in multiple-cat households than traditional approaches alone. A PHTE may be an effective and practical tool that veterinarians can use in feline weight-loss strategies. However, best practices need to be established to maximize adoption and increase impact. Devices should be affordable and easy to operate, providing useful data for caregivers that are immediate, simple and actionable. More research is needed to determine how technology can best be leveraged to deal with pet weight management and other major challenges in pet healthcare. Technology will ultimately help drive a shift from episodic to continuous monitoring and from reactive to proactive precision medicine.

## Supplemental Material

Table S1 Click here for additional data file. Meeting specific weight loss challenges with technology

Table S2 Click here for additional data file. Previous owner experience

Table S3 Click here for additional data file. Owner perception of WLP

Table S4 Click here for additional data file. Owner comments on the PHTE

Table S5 Click here for additional data file. Owner comments on cat behavior

Diary Click here for additional data file. technology group

Diary Click here for additional data file. traditional group

Questionnaire Click here for additional data file. owner post-WLP

Questionnaire Click here for additional data file. owner pre-WLP
