# Supplementary material for: Technology-enhanced weight-loss program in multiple-cat households: a randomized controlled trial
Source: J Feline Med Surg. 2021 Oct 21;24(8):726–38. doi: 10.1177/1098612X211044412 (PMC9315194; doi:10.1177/1098612X211044412)
Supplement: Table S1 [file sj-docx-1-jfm-10.1177_1759720X211043977.docx]

Table S1Meeting specific weight loss challenges with technology

| Weight loss challenge | Technology Solution | Considerations |
| --- | --- | --- |
| Frequent reassessment and tracking of body weight | Digital scale | Accuracy, active vs passive weighing, client & pet friendly, recording of weight (manual diary or automatic app) |
| Delivering the correct amount of food, multiple times a day, to the correct pet | Smart feeders | Accuracy, wet vs dry food, client & pet friendly, need to dispense less than 1/2 cup when using an Rx food with average Cal of around 300-350/cup |
| Monitoring and awareness of pet activity | Activity monitor | Cat tolerance, need a collar or harness, light weight, long battery life, good app/user interface, units - activity counts vs calories vs inactive behaviors, other cat data |
| Managing feeding-related behaviors &owner engagement | Motion/sound activated webcam | Functionality, ease of use, security, privacy |
| Getting cats to exercise (indoor-only cats especially) | Smart exercise device | Ease of use, cat tolerance and interest, most still require time from pet parent, hard to ‘prescribe’ fitness session for a cat, none are patient specific yet, AI powered |
| Proper nutrition - what to feed, how much to feed, how to get it | Advanced food &online resources | Advanced/prescriptions foods, online non-branded nutritional calculators, online nutritional content database of pet foods, body condition score, easier access to food |
| Keeping track of metrics | Digital/online diary | Ease of use, easily share data with vet, consolidated, included with app that comes with other devices |
