# Supplementary material for: Technology-enhanced weight-loss program in multiple-cat households: a randomized controlled trial
Source: J Feline Med Surg. 2021 Oct 21;24(8):726–38. doi: 10.1177/1098612X211044412 (PMC9315194; doi:10.1177/1098612X211044412)
Supplement: Table S2 [file sj-docx-2-jfm-10.1177_1759720X211043977.docx]

## Table S2Previous owner experience (n=13)

| Category | Response (number of owners) |
| --- | --- |
| Technology ability - basic use of computer, internet, smartphones, and smart devices | proficient, advanced or expert (13) *  none, basic, or minimal (0) |
| Previous use of home pet devices | smart feeder (5), pet treat camera (3), home pet scale (1), activity monitor (1) |
| Number of daily feedings | twice (6), three times (5), leave food out (2) |
| Other pets in household | dog (7), turtle (1), rabbit (1) |
| Owner description of cat’s weight** | mildly overweight (13), moderately overweight (5), obese (4) |
| Previous attempted weight loss strategies | decreased food amount (10), diet change (5), supervised feeding (5), increased activity (4) |
| Weight loss challenges | cat stealing other cat’s food (11), knowing activity level and target exercise amount (10), methods to increase activity (8), figuring out ideal weight (8), monitoring weight loss progress (7), other people feeding (6), overfeeding due to begging (5), choosing diet (5), convenience of leaving out food (4), knowing/measuring amount (3), expense of weight loss diet (3) |

*Representing each of the four technology sub-categories; **excluding normal weight cats
