# Supplementary material for: Technology-enhanced weight-loss program in multiple-cat households: a randomized controlled trial
Source: J Feline Med Surg. 2021 Oct 21;24(8):726–38. doi: 10.1177/1098612X211044412 (PMC9315194; doi:10.1177/1098612X211044412)
Supplement: Table S3 [file sj-docx-3-jfm-10.1177_1759720X211043977.docx]

## Table S3 Owner perception of WLP (n=10)

| Statement | Level of Agreement  (# of owners) |
| --- | --- |
| My cat’s feeding recommendations were clear and easy to follow | Strong (7), Somewhat (3) |
| I am more aware of my cat’s needs | Strong (6), Somewhat (4) |
| I learned something about cat weight loss | Strong (1), Somewhat (8) |
| I felt empowered to impact the health and well-being of my cat | Strong (6), Somewhat (3) |
| I feel my cat is healthier as a result of the weight management program | Strong (6), Somewhat (3) |
| My cat’s appearance has improved | Strong (3), Somewhat (9) |
| I have a greater appreciation with my vet | Strong (3), Somewhat (6) |
| I am pleased with my cat’s results in the weight management program | Strong (6), Somewhat (2) |
| I feel closer to my cat | Strong (1), Somewhat (4) |
| My cat’s relationship with my other cat has improved | Strong (1), Somewhat (2) |
