# Supplementary material for: Technology-enhanced weight-loss program in multiple-cat households: a randomized controlled trial
Source: J Feline Med Surg. 2021 Oct 21;24(8):726–38. doi: 10.1177/1098612X211044412 (PMC9315194; doi:10.1177/1098612X211044412)
Supplement: Table S4 [file sj-docx-4-jfm-10.1177_1759720X211043977.docx]

## Table S4 Owner comments on the PHTE

| ‘Relatively easy to implement.’ |
| --- |
| ‘Overall I was very happy with how much weight my cat lost. I was very impressed at how the technology is helpful in weight management.’ |
| ‘Once I figured out all of software easy to use. Some of apps didn’t cooperate as smoothly as I felt they should. Overall happy with weight loss of both cats.’ |
| ‘This weight loss program worked the best for my cats. I have tried others in the past. I stopped using the equipment a bit ago…and I’ve noticed one of my cats is starting to gain a bit of weight back.’ |
| ‘No complaints...everything is working great!’ |
| ‘Need to have a company that does the support for all the equipment to make it more user friendly.’ |
| ‘I strongly feel that the feeders where the most important part of this weight loss program above any of the equipment used.’ |
| ‘Timid around the feeder but figuring it out.’ |
| ‘The design makes it hard for one cat to steal food from the other.’ |
| ‘So much more calm, no pestering for food.’ |
| ‘My cat feels very safe and less stressed since able to leave food in feeder without other cat being able to take it.’ |
| ‘The youngest cat would ALWAYS steal food from the older cat’s feeder before the door would close even on the fastest setting.’ |
| ‘The [feeder] with the scale was troublesome at times and had to frequently be reset for the cat’s information.’ |
| ‘I don’t think the [smart feeder] is correctly recording. [Cat] is recorded as eating once - 16 grams. In fact she was fed 3 times (16 grams each) and ate it all… sometimes the weight recorded is a bit higher. Suspect that the zeroing was not accurate as it is recording a few grams over. Also might reflect the normal variation around the set weight.’ |
| ‘Troubleshooting the 1 [feeder] this week. Not sure why this one is giving me trouble…[feeder] with the scale seems more problemsome for me than the original one.’ |
| ‘The [feeder] needs to be able to store the data when it loses connection.’ |
| ‘The [feeder] hub needs to be wireless.’ |
| ‘The [activity monitor] is EVERYTHING!...The [activity monitor] is my favorite piece of equipment. I need to get these [activity monitors].’ |
| ‘[Cat] was limping.  Checked in on the [activity monitor platform], noted [cat] activity is down!  Something I don't think I would have noted at home.  It was very subtle!’ |
| ‘Interesting to see that [cat 1] moved a lot more than [cat 2] in a day. While the [activity monitor] is interesting, don't see the value. The number of steps doesn't relate to anything.’ |
| ‘Helped me to know when the cats were active and to see that she was getting more active as she lost weight.’ |
| ‘The [activity monitor] will only become useful if it has comparable fitness measures applicable to cats, not dogs.’ |
| ‘The design of the tracker itself was great! Light and low profile, the cats did not mind it.’ |
| ‘Not happy about the new collar but adjusting…I think that cats scratching their collar likely increases the number of steps.’ |
| ‘Between the [pet treat camera] and [activity monitor], I love how I can monitor the kitties away from home! Kitties come running to the [pet treat camera] when I say hi!’ |
| ‘‘I love the night vision camera, it was VERY informative. I was not aware of how much my cats eat and play at night.’ |
| ‘While it let me see that the cats were eating each other's food, that was something I knew.’ |
| ‘Not big on treats so no interest in the pet camera dispensing treats. Not using the [pet treat camera] as it is glitchy.  Also, [cat] does not take the type of treats that are dispensed.  Mostly gets the dogs attention.’ |
| ‘I find having the baby scale helpful; immediate feedback’ |
| ‘Having a scale at home was very important to track the success of my cat's diet. I have already purchased one to make sure that she does not gain any of the weight back.’ |
| ‘Kitties love napping on the scale!’ |
| ‘Incorporate [scale] into bed, under litter box, or in front of feeder to make passive.’ |
