# Supplementary material for: Technology-enhanced weight-loss program in multiple-cat households: a randomized controlled trial
Source: J Feline Med Surg. 2021 Oct 21;24(8):726–38. doi: 10.1177/1098612X211044412 (PMC9315194; doi:10.1177/1098612X211044412)
Supplement: Table S5 [file sj-docx-5-jfm-10.1177_1759720X211043977.docx]

## Table S5 Owner comments on cat behavior

| ‘Mostly normal, increased in begging and getting into human food, activity is up.’ |
| --- |
| ‘Begging has increased, initiating play with other cat, more active, finishing food quickly.’ |
| ‘[Cat] always seems starving before her meals. [Cat] seems more lively now that spring has sprung. She is sleeping less and moving more. She is also more affectionate.’ |
| ‘[Cat] is ravenous. Seems desperate around feeding time to get food. Continues to eat everything offered.’ |
| ‘[Cat’s] energy is higher than I have seen in a long time. She is playing more.’ |
| ‘Never been a high energy cat, fairly similar to usual behavior. strong appetite. will finish food but more slowly than usual, not as enthusiastic about mealtime.’ |
| ‘Very affectionate, more active than usual.’ |
| ‘Still often not finishing own food during the day, though usually will do so overnight.’ |
| ‘Standard mischievous behavior around mealtime, has been waking up in the middle of the night wanting to be fed.’ |
| ‘Playing a bit more, still primarily interested in low-effort games and toys.’ |
| ‘Some begging, consistently goes after other cats food, showing more aggression towards the other cat near food time’ |
| ‘Very energetic and playful. He is interested in food and they eat everything usually by noon.’ |
| ‘He seems to be looking for food. Night zooming and tapping for food.’ |
| ‘No real change. Still same energy level and contentment. She eats her food in the morning and the naps. No begging.’ |
| ‘They both tend to eat the food faster in the morning.’ |
| ‘Hasn't changed much, appetite is about the same, his begging is increased, but not annoying like other cat.’ |
| ‘More energy, very happy and loving, appetite about the same, will eat when given food, but doesn't seek you out to beg or demand, very opportunistic.’ |
