# Supplementary material for: Technology-enhanced weight-loss program in multiple-cat households: a randomized controlled trial
Source: J Feline Med Surg. 2021 Oct 21;24(8):726–38. doi: 10.1177/1098612X211044412 (PMC9315194; doi:10.1177/1098612X211044412)
Supplement: Diary [file sj-pdf-6-jfm-10.1177_1759720X211043977.pdf]

Diary for Oldest Cat

Participant ID (letter and number): ________________ (Please fill out twice weekly)

Week	Date 1<br>(for body weight)	Home scale weight 1<br>(x.xx kg) (1kg=2.2*lb)	Date 2<br>(for body weight)	Home scale weight 2<br>(x.xx kg) (1kg=2.2*lb)	Hills Metabolic Amount fed<br>(grams /day)	Other treats, wet food, dry food fed<br>(kcalories /day)	Cat behavior observations<br>(with specific attention to energy, happiness, appetite, begging)	Pet health technology ecosystem comments	General Comments
1									
2									
3									
4									
5									
6									
7									
8									
9									
10									
11									
12									
13									
14									

Diary for Youngest Cat

Participant ID (letter and number): ________________ (Please fill out twice weekly)

Week	Date 1 (for body weight)	Home scale weight 1 (x.xx kg) (1kg=2.2*lb)	Date 2 (for body weight)	Home scale weight 2 (x.xx kg) (1kg=2.2*lb)	Hills Metabolic Amount fed (grams /day)	Other treats, wet food, dry food fed (kcalories /day)	Cat behavior observations (with specific attention to energy, happiness, appetite, begging)	Pet health technology ecosystem comments	General Comments
1									
2									
3									
4									
5									
6									
7									
8									
9									
10									
11									
12									
13									
14									

Diary for Middle Cat

Participant ID (letter and number): ________________ (Please fill out twice weekly)

Week	Date 1<br>(for body weight)	Home scale weight 1<br>(x.xx kg) (1kg=2.2*lb)	Date 2<br>(for body weight)	Home scale weight 2<br>(x.xx kg) (1kg=2.2*lb)	Hills Metabolic Amount fed<br>(grams /day)	Other treats, wet food, dry food fed<br>(kcalories /day)	Cat behavior observations<br>(with specific attention to energy, happiness, appetite, begging)	Pet health technology ecosystem comments	General Comments
1									
2									
3									
4									
5									
6									
7									
8									
9									
10									
11									
12									
13									
14									
