# Supplementary material for: Technology-enhanced weight-loss program in multiple-cat households: a randomized controlled trial
Source: J Feline Med Surg. 2021 Oct 21;24(8):726–38. doi: 10.1177/1098612X211044412 (PMC9315194; doi:10.1177/1098612X211044412)
Supplement: Diary [file sj-pdf-7-jfm-10.1177_1759720X211043977.pdf]

Diary for Oldest Cat

Participant ID (letter and number):

Week	Date	Weight (from vet) (x.xx kg) (1kg=2.2*lb)	Hills Metabolic Amount fed (grams /day)	Other treats, wet food, dry food fed (kcalories /day)	Cat behavior observations (with specific attention to energy, happiness, appetite, begging)	General Comments
1						
2						
3						
4						
5						
6						
7						
8						
9						
10						
11						
12						
13						
14						

Diary for Youngest Cat

Participant ID (letter and number):

Week	Date	Weight (from vet) (x.xx kg) (1kg=2.2*lb)	Hills Metabolic Amount fed (grams /day)	Other treats, wet food, dry food fed (kcalories /day)	Cat behavior observations (with specific attention to energy, happiness, appetite, begging)	General Comments
1						
2						
3						
4						
5						
6						
7						
8						
9						
10						
11						
12						
13						
14						

Diary for Middle Cat

Participant ID (letter and number):

Week	Date	Weight (from vet) (x.xx kg) (1kg=2.2*lb)	Hills Metabolic Amount fed (grams /day)	Other treats, wet food, dry food fed (kcalories /day)	Cat behavior observations (with specific attention to energy, happiness, appetite, begging)	General Comments
1						
2						
3						
4						
5						
6						
7						
8						
9						
10						
11						
12						
13						
14						
