# Supplementary material for: Technology-enhanced weight-loss program in multiple-cat households: a randomized controlled trial
Source: J Feline Med Surg. 2021 Oct 21;24(8):726–38. doi: 10.1177/1098612X211044412 (PMC9315194; doi:10.1177/1098612X211044412)
Supplement: Questionnaire [file sj-pdf-8-jfm-10.1177_1759720X211043977.pdf]

English

General

"The Use of a Pet Health Technology Ecosystem (PHTE) in a Weight Management Program (WMP) in Multiple Cat Households in the US & Canada"

Post cat weight management program (WMP) eSurvey for owners

You have just completed a 14-week cat weight loss program. Now we would like to hear your thoughts regarding this program. This will help us to evaluate the value of a pet health technology ecosystem (PHTE) as a tool in battling cat obesity. We thank you for you participation in the study.

What is your participant ID?
(letter and number, e.g. A1)

Involved Cats

How many cats do you have in your household?

- I have 1 cat
- I have 2 cats
- I have 3 cats
- I have 4 cats
- I have 5 cats
- I have 6 cats
- I have 7 cats
- I have 8 or more cats

Is your **OLDEST cat** part of the weight loss program?
(if your cats are the same age or you are unsure of their age, pick the heaviest one as the oldest)

- Yes
- No

Is your **YOUNGEST cat** part of the weight loss program?

- Yes

◯ No

Is your **MIDDLE cat** part of the weight loss program?

◯ Yes
◯ No

WMP Satisfaction Oldest Cat

Please fill out the following information regarding your **OLDEST cat** and the **weight management program**....

For each statement, check whether you strongly agree, somewhat agree, somewhat disagree, or strongly disagree. You may refuse to answer.

	Agree Strongly	Agree Somewhat	Neither Agree or Disagree	Disagree Somewhat	Disagree Strongly	Don't Know or Refuse
My cat's feeding recommendations were clear and easy to follow.	◯	◯	◯	◯	◯	◯
I learned something new about weight loss in cats.	◯	◯	◯	◯	◯	◯
I am more aware of my cat's needs.	◯	◯	◯	◯	◯	◯
I felt empowered to impact the health and well-being of my cat.	◯	◯	◯	◯	◯	◯
I feel a sense of accomplishment.	◯	◯	◯	◯	◯	◯
I feel closer to my cat.	◯	◯	◯	◯	◯	◯
My cat's relationship with my other cat has improved.	◯	◯	◯	◯	◯	◯
My cat's appearance has improved.	◯	◯	◯	◯	◯	◯
I feel my cat is healthier as a result of the weight management program.	◯	◯	◯	◯	◯	◯
I am pleased with my cat's results in the weight management program.	◯	◯	◯	◯	◯	◯
I have a greater appreciation for my veterinarian.	◯	◯	◯	◯	◯	◯

WMP Satisfaction Youngest Cat

Please fill out the following information regarding your **YOUNGEST cat** and the **weight management program...**

For each statement, check whether you strongly agree, somewhat agree, somewhat disagree, or strongly disagree. You may refuse to answer.

	Agree Strongly	Agree Somewhat	Neither Agree or Disagree	Disagree Somewhat	Disagree Strongly	Don't Know or Refuse
My cat's feeding recommendations were clear and easy to follow.	○	○	○	○	○	○
I learned something new about weight loss in cats.	○	○	○	○	○	○
I am more aware of my cat's needs.	○	○	○	○	○	○
I felt empowered to impact the health and well-being of my cat.	○	○	○	○	○	○
I feel a sense of accomplishment.	○	○	○	○	○	○
I feel closer to my cat.	○	○	○	○	○	○
My cat's relationship with my other cat has improved.	○	○	○	○	○	○
My cat's appearance has improved.	○	○	○	○	○	○
I feel my cat is healthier	○	○	○	○	○	○
I am pleased with my cat's results	○	○	○	○	○	○
I have a greater appreciation for my veterinarian.	○	○	○	○	○	○

WMP Satisfaction Middle Cat

Please fill out the following information regarding your **MIDDLE cat** and the **weight management program...**

For each statement, check whether you strongly agree, somewhat agree, somewhat disagree, or strongly disagree. You may refuse to answer.

	Agree Strongly	Agree Somewhat	Neither Agree or Disagree	Disagree Somewhat	Disagree Strongly	Don't Know or Refuse
My cat's feeding recommendations were clear and easy to follow.	○	○	○	○	○	○
I learned something new about weight loss in cats.	○	○	○	○	○	○
I am more aware of my cat's needs.	○	○	○	○	○	○

	Agree Strongly	Agree Somewhat	Neither Agree or Disagree	Disagree Somewhat	Disagree Strongly	Don't Know or Refuse
I felt empowered to impact the health and well-being of my cat.	○	○	○	○	○	○
I feel a sense of accomplishment.	○	○	○	○	○	○
I feel closer to my cat.	○	○	○	○	○	○
My cat's relationship with my other cat has improved.	○	○	○	○	○	○
My cat's appearance has improved.	○	○	○	○	○	○
I feel my cat is healthier	○	○	○	○	○	○
I am pleased with my cat's results	○	○	○	○	○	○
I have a greater appreciation for my veterinarian.	○	○	○	○	○	○

WMP notes

Any other comments regarding your satisfaction with the weight management program? (e.g. ease of implementation, outcome, program management/communication of veterinarian, etc.)

Did you receive a pet health technology ecosystem (PHTE)?

- ○ Yes
- ○ No

PHTE

The following questions pertain to your experience using the pet health technology ecosystem (PHTE) to assist with weight loss.

How effective do you think the **PHTE** can be in a feline weight management program?

- ○ Extremely effective
- ○ Very effective
- ○ Moderately effective

- Slightly effective
- Not effective at all

How satisfied were you with the **PHTE** overall?

- Extremely satisfied
- Very satisfied
- Moderately satisfied
- Slightly satisfied
- Not satisfied at all

Would you continue to use a **PHTE** in conjunction with a weight management program?

- No
- Yes

How likely is it that you would recommend a **PHTE** to a friend or colleague?

Not at all likely ... Extremely likely

0 ○ 1 ○ 2 ○ 3 ○ 4 ○ 5 ○ 6 ○ 7 ○ 8 ○ 9 ○ 10 ○

What is the most you would pay for the **PHTE**?

- Nothing, I wouldn't buy it
- $1 - $100
- $101 - $200
- $201 - $300
- $301 - $400
- $401 - $500
- $501 - $600
- $601+

Please rate your satisfaction with the following regarding the features and functions of the **SureFeed connect smart feeder**.

	Very Satisfied	Moderately Satisfied	Slightly Satisfied	Less Satisfied	Not Satisfied	Not Applicable
Device (hardware) design	○	○	○	○	○	○
Device setup	○	○	○	○	○	○
Device use	○	○	○	○	○	○
Accuracy of feed amount dispensed	○	○	○	○	○	○
Usefulness	○	○	○	○	○	○
Customer service	○	○	○	○	○	○

How likely is it that you would recommend a **smart feeder (like SureFeed connect)** to a friend or colleague?

Not at all likely ... Extremely likely

0 ◯ 1 ◯ 2 ◯ 3 ◯ 4 ◯ 5 ◯ 6 ◯ 7 ◯ 8 ◯ 9 ◯ 10 ◯

Please rate your satisfaction with the following regarding the features and functions of the **FitBark activity monitor**.

	Very Satisfied	Moderately Satisfied	Slightly Satisfied	Less Satisfied	Not Satisfied	Not Applicable
Device (hardware) design	◯	◯	◯	◯	◯	◯
Device setup	◯	◯	◯	◯	◯	◯
Device use	◯	◯	◯	◯	◯	◯
App set pp	◯	◯	◯	◯	◯	◯
App use	◯	◯	◯	◯	◯	◯
Accuracy of activity counts	◯	◯	◯	◯	◯	◯
Usefulness	◯	◯	◯	◯	◯	◯
Customer service	◯	◯	◯	◯	◯	◯

How likely is it that you would recommend an **activity monitor (like FitBark)** to a friend or colleague?

Not at all likely ... Extremely likely

0 ◯ 1 ◯ 2 ◯ 3 ◯ 4 ◯ 5 ◯ 6 ◯ 7 ◯ 8 ◯ 9 ◯ 10 ◯

Please rate your satisfaction with the following regarding the features and functions of the **home scale**.

	Very Satisfied	Moderately Satisfied	Slightly Satisfied	Less Satisfied	Not Satisfied	Not Applicable
Device (hardware) design	◯	◯	◯	◯	◯	◯
Device setup	◯	◯	◯	◯	◯	◯
Device use	◯	◯	◯	◯	◯	◯
Accuracy of weight	◯	◯	◯	◯	◯	◯
Usefulness	◯	◯	◯	◯	◯	◯

How likely is it that you would recommend a **home scale** to a friend or colleague?

Not at all likely ... Extremely likely

0	1	2	3	4	5	6	7	8	9	10
○	○	○	○	○	○	○	○	○	○	○

Please rate your satisfaction with the following regarding the features and functions of the **Petcube Bites.**

	Very Satisfied	Moderately Satisfied	Slightly Satisfied	Less Satisfied	Not Satisfied	Not Applicable
Device (hardware) design	○	○	○	○	○	○
Device set up	○	○	○	○	○	○
Device use	○	○	○	○	○	○
App set up	○	○	○	○	○	○
App use	○	○	○	○	○	○
Usefulness	○	○	○	○	○	○
Customer support	○	○	○	○	○	○

How likely is it that you would recommend a **webcam/treat dispenser (like Petcube Bites)** to a friend or colleague?

Not at all likely — Extremely likely

0	1	2	3	4	5	6	7	8	9	10
○	○	○	○	○	○	○	○	○	○	○

Please comment on the strength/weaknesses of the **PHTE**. How would you improve the **PHTE** and/or what other devices or software would you add?

Any other comments?

Powered by Qualtrics
