# Supplementary material for: Technology-enhanced weight-loss program in multiple-cat households: a randomized controlled trial
Source: J Feline Med Surg. 2021 Oct 21;24(8):726–38. doi: 10.1177/1098612X211044412 (PMC9315194; doi:10.1177/1098612X211044412)
Supplement: Questionnaire [file sj-pdf-9-jfm-10.1177_1759720X211043977.pdf]

English

Background Information

"The Use of a Pet Health Technology Ecosystem (PHTE) in a Weight Management Program (WMP) in Multiple Cat Households in the US & Canada"

Initial eSurvey for owners

Welcome to the study! You are helping us research the use of a PHTE in a cat weight management program. We would first like to get some background information about you. We thank you for joining us in this project.

What is your participant ID?
(letter and number, e.g. A1)

What is your age (in years)?

Which gender do you most identify with?

- ◯ Male
- ◯ Female
- ◯ Other
- ◯ Prefer not to disclose

What country do you live in?

- ◯ USA
- ◯ Canada
- ◯ Other (please specify)

How would you rate your technology use/abilities in the following categories?

	Tech Illiterate					Tech-Savvy
	**None**: No use or experience	**Minimal**: Rare use or just started learning	**Basic**: I have some experience, use it every now and then, but might need help	**Proficient**: I use it frequently and can usually figure it out without assistance	**Advanced**: I use it all the time and usually teach others how to do this	**Expert**: I am a professional or this is part of my career
**Basic computer use** (e.g. word, excel, audio/visual media)	○	○	○	○	○	○
**Internet use** (e.g. email, web surfing, social media)	○	○	○	○	○	○
**Smartphone use** (e.g. apps)	○	○	○	○	○	○
**Smart device use** (e.g. TV, Blu-ray/media players, Amazon Echo, Google Home, Phillip Hue lights, pet feeders)	○	○	○	○	○	○

Which of the following do you currently have or have used previously? (select all that apply)

- ☐ [ ] Smart feeder (please specify which)
- ☐ [ ] Activity monitor (please specify which)
- ☐ [ ] Home pet scale (please specify which)
- ☐ [ ] Treat dispenser (please specify which)
- ☐ [ ] Pet webcam (please specify which)
- ☐ [ ] Other device(s) (please specify)
- ☐ None of these

How many cats do you have in your household?

- ○ I have 1 cat
- ○ I have 2 cats
- ○ I have 3 cats
- ○ I have 4 cats
- ○ I have 5 cats
- ○ I have 6 cats
- ○ I have 7 cats
- ○ I have 8 or more cats

Do you have any other pets in your household?

- ◯ No
- ◯ Yes (please specify)

[ ]

Cat Info

Please answer the following question regarding you **oldest cat**...

What is the age (in years) of your **oldest cat**?

What is the sex of your **oldest cat**?

- ◯ Male Intact
- ◯ Male Castrated
- ◯ Female Intact
- ◯ Female Spayed

What breed is your **oldest cat**?

Where does your **oldest cat** spend most of the time?

- ◯ Indoor
- ◯ Outdoor
- ◯ Indoor/Outdoor

Please list below the brands and product names (if applicable) and amounts of ALL foods and treats that you give to your **oldest cat** in a day?
(select all that apply)

- ☐ Wet food
- ☐ Dry food
- ☐ Treats

☐ Special needs (e.g. kidney diet)

Do you give any dietary supplements to your **oldest cat**? (e.g. vitamins, glucosamine, fatty acids, or any other supplements)

- ◯ No
- ◯ Yes (please list brands and amounts)

How many times do you usually feed your **oldest cat** in a day?

- ◯ Once per day
- ◯ Twice per day
- ◯ Three times per day
- ◯ Four times per day
- ◯ Five times per day
- ◯ Six times a day
- ◯ Seven or more times a day
- ◯ I leave food out all day

Please answer the following question regarding you **youngest cat**...

What is the age (in years) of your **youngest cat**?

What is the sex of your **youngest cat**?

- ◯ Male Intact
- ◯ Male Castrated
- ◯ Female Intact
- ◯ Female Spayed

What breed is your **youngest cat**?

Where does your **youngest cat** spend most of the time?

- ◯ Indoor
- ◯ Outdoor
- ◯ Indoor/Outdoor

Please list below the brands and product names (if applicable) and amounts of ALL foods and treats that you give to your **youngest cat** in a day?
(select all that apply)

- ☐ Wet food
- ☐ Dry food
- ☐ Treats
- ☐ Special needs (e.g. kidney diet)

Do you give any dietary supplements to your **youngest cat**? (e.g. vitamins, glucosamine, fatty acids, or any other supplements)

- ○ No
- ○ Yes (please list brands and amounts)

How many times do you usually feed your **youngest cat** in a day?

- ○ Once per day
- ○ Twice per day
- ○ Three times per day
- ○ Four times per day
- ○ Five times per day
- ○ Six or more times a day
- ○ Seven or more times a day
- ○ I leave food out all day

Please answer the following question regarding you **middle cat**...

What is the age (in years) of your **middle cat**?

What is the sex of your **middle cat**?

- ○ Male Intact
- ○ Male Castrated
- ○ Female Intact

○ Female Spayed

What breed is your **middle cat**?

[ ]

Where does your **middle cat** spend most of the time?

○ Indoor
○ Outdoor
○ Indoor/Outdoor

Please list below the brands and product names (if applicable) and amounts of ALL foods and treats that you give to your **middle cat** in a day?
(select all that apply)

☐ Wet food
[ ]
☐ Dry food
[ ]
☐ Treats
[ ]
☐ Special needs (e.g. kidney diet)
[ ]

Do you give any dietary supplements to your **middle cat**? (e.g. vitamins, glucosamine, fatty acids, or any other supplements)

○ No
○ [ ] Yes (please list brands and amounts)

How many times do you usually feed your **middle cat** in a day?

○ Once per day
○ Twice per day
○ Three times per day
○ Four times per day
○ Five times per day
○ Six or more times a day
○ Seven or more times a day
○ I leave food out all day

If you feed by volume, what size measuring device do you use?

If you feed tinned/canned food, what size tins/cans?

Weight Loss History

How would you describe your **oldest cat's** weight?

- ◯ Obese
- ◯ Moderately overweight
- ◯ Mildly overweight
- ◯ Ideal weight
- ◯ Mildly underweight
- ◯ Moderately underweight
- ◯ Emaciated

How would you describe your **youngest cat's** weight?

- ◯ Obese
- ◯ Moderately overweight
- ◯ Mildly overweight
- ◯ Ideal weight
- ◯ Mildly underweight
- ◯ Moderately underweight
- ◯ Emaciated

How would you describe your **middle cat's** weight?

- ◯ Obese
- ◯ Moderately overweight
- ◯ Mildly overweight
- ◯ Ideal weight
- ◯ Mildly underweight
- ◯ Moderately underweight
- ◯ Emaciated

For each statement, check whether you strongly agree, somewhat agree, somewhat disagree, or strongly disagree. You may refuse to answer.

	Agree Strongly	Agree Somewhat	Disagree Somewhat	Disagree Strongly	Don't Know or Refuse
Having my cat at an ideal weight is important to me.	○	○	○	○	○
I believe weight loss impacts my cat's health.	○	○	○	○	○

What weight loss strategies have you previously tried prior to this weight loss program?
(select all that apply)

- ☐ I have not tried any weight management strategies
- ☐ Decreased amount of food and/or treats
- ☐ Diet change (e.g. weight loss diet)
- ☐ Increased feeding frequency
- ☐ Increased activity
- ☐ Separated/supervised feedings (for multi cat households)
- ☐ Other/Comments

Prior to this weight loss program, did you use any of the following to increase your cat(s) activity?

- ☐ Leash walks
- ☐ Supervised outside time
- ☐ Toys
- ☐ Laser pointer
- ☐ Smart device
- ☐ Other (please specify)

What have been the biggest challenges in trying to get your cat(s) to ideal weight? (select all that apply)

Diet Challenges:

- ☐ Knowing and/or measuring ideal amount to feed
- ☐ Convenience of leaving out constant supply of food
- ☐ Overfeeding due to begging or other behaviors
- ☐ Other family members/people not following the weight management plan (e.g. gives extra food/treats)
- ☐ One cat eating other cat's food
- ☐ Choosing a good weight management diet

- [ ] Expense of weight loss diet

Activity Challenges:

- [ ] Finding effective methods to increase my cats activity
- [ ] Knowing how active my cat is and how much exercise he/she should get

Other Challenges:

- [ ] Figuring out the ideal weight for my cat
- [ ] Monitoring weight loss progress
- [ ] Other (please specify)

Human-Animal Bond

The following questions aim at measuring the human-animal bond between you and your cat(s).

Lexington Attachment to Pets Scale

Please tell us whether you agree or disagree with some very brief statements about your cat(s) on the weight loss program. For each statement, check whether you strongly agree, somewhat agree, somewhat disagree, or strongly disagree. You may refuse to answer.

	Agree Strongly	Agree Somewhat	Disagree Somewhat	Disagree Strongly	Don't Know or Refuse
My pet means more to me than any of my friends.	○	○	○	○	○
Quite often I confide in my pet.	○	○	○	○	○
I believe that pets should have the same rights and privileges as family members.	○	○	○	○	○
I believe my pet is my best friend.	○	○	○	○	○
Quite often, my feelings toward people are affected by the way they react to my pet.	○	○	○	○	○
I love my pet because he/she is more loyal to me than most of the people in my life.	○	○	○	○	○
I enjoy showing other people pictures of my pet.	○	○	○	○	○
I think my pet is just a pet.	○	○	○	○	○

	Agree Strongly	Agree Somewhat	Disagree Somewhat	Disagree Strongly	Don't Know or Refuse
I love my pet because it never judges me.	○	○	○	○	○
My pet knows when I'm feeling bad.	○	○	○	○	○
I often talk to other people about my pet.	○	○	○	○	○
My pet understands me.	○	○	○	○	○

Human-Animal Bond Continuation

Lexington Attachment to Pets Scale Continuation

Please tell us whether you agree or disagree with some very brief statements about your cat(s) on the weight loss program. For each statement, check whether you strongly agree, somewhat agree, somewhat disagree, or strongly disagree. You may refuse to answer.

	Agree Strongly	Agree Somewhat	Disagree Somewhat	Disagree Strongly	Don't Know or Refuse
I believe that loving my pet helps me stay healthy.	○	○	○	○	○
Pets deserve as much respect as humans do.	○	○	○	○	○
My pet and I have a very close relationship.	○	○	○	○	○
I would do almost anything to take care of my pet.	○	○	○	○	○
I play with my pet quite often.	○	○	○	○	○
I consider my pet to be a great companion.	○	○	○	○	○
My pet makes me feel happy.	○	○	○	○	○
I feel that my pet is a part of my family.	○	○	○	○	○
I am not very attached to my pet.	○	○	○	○	○
Owning a pet adds to my happiness.	○	○	○	○	○
I consider my pet to be a friend.	○	○	○	○	○

Powered by Qualtrics
